# Multicentre, retrospective study of the efficacy and safety of nivolumab for recurrent and metastatic salivary gland carcinoma

**DOI:** 10.1038/s41598-020-73965-6

**Published:** 2020-10-12

**Authors:** Kazutomo Niwa, Daisuke Kawakita, Toshitaka Nagao, Hideaki Takahashi, Takashi Saotome, Masashi Okazaki, Keisuke Yamazaki, Isaku Okamoto, Hideaki Hirai, Natsuki Saigusa, Chihiro Fushimi, Tatsuo Masubuchi, Kouki Miura, Shin-ichi Okazaki, Hirooki Matsui, Takuro Okada, Sho Iwaki, Takashi Matsuki, Kenji Hanyu, Kiyoaki Tsukahara, Nobuhiko Oridate, Yuichiro Tada

**Affiliations:** 1 Department of Head and Neck Oncology and Surgery, International University of Health and Welfare, Mita Hospital, Tokyo, 108-8329 Japan; 2 Department of Otorhinolaryngology, Head and Neck Surgery, Yokohama City University, School of Medicine, Yokohama, Kanagawa 236-0004 Japan; 3 Department of Otorhinolaryngology, Head and Neck Surgery, Nagoya City University Graduate School of Medical Sciences, Nagoya, 467-8602 Japan; 4 Department of Anatomic Pathology, Tokyo Medical University, Tokyo, 160-0023 Japan; 5 Division of Medical Oncology, Matsudo City Hospital, Chiba, 270-2252 Japan; 6 Department of Otorhinolaryngology, Head and Neck Surgery, Nihonkai General Hospital, Yamagata, 998-8501 Japan; 7 Department of Otolaryngology Head and Neck Surgery, Niigata University Graduate School of Medical and Dental Sciences, Niigata, 951-8520 Japan; 8 Department of Otorhinolaryngology Head and Neck Surgery, Tokyo Medical University, Tokyo, 160-0023 Japan; 9 Department of Otorhinolaryngology, Head and Neck Surgery, Kitasato University School of Medicine, Sagamihara, 252-0375 Japan

**Keywords:** Cancer, Biomarkers, Diseases, Medical research, Oncology, Risk factors

## Abstract

Although immune-checkpoint inhibitors (ICIs) are effective against various cancers, little is known regarding their role in salivary gland carcinoma (SGC) treatment. Therefore, we evaluated the efficacy and safety of nivolumab monotherapy in patients with recurrent and/or metastatic SGC. In this multicentre retrospective study, nivolumab (240 mg) was administered every 2 weeks. The overall response rate (ORR), progression-free survival (PFS), overall survival (OS), and safety were examined; the correlation between treatment outcomes and clinicopathological factors was analysed. Twenty-four patients were enrolled; the most common histopathology was salivary duct carcinoma. Eleven tumours were PD-L1-positive; no tumour was microsatellite instability-high. The ORR was 4.2%, and the median PFS and OS were 1.6 and 10.7 months, respectively. One patient continued nivolumab for 28 months without disease progression. One patient showed grade 4 increase in creatine phosphokinase levels and grade 3 myositis. Biomarker analysis revealed significantly increased OS in patients with performance status of 0; modified Glasgow prognostic score of 0; low neutrophil-to-lymphocyte ratio, lactate dehydrogenase, and C-reactive protein; and high lymphocyte-to-monocyte ratio and in patients who received systemic therapy following nivolumab. Although nivolumab’s efficacy against SGC was limited, some patients achieved long-term disease control. Further studies are warranted on ICI use for SGC.

## Introduction

Salivary gland carcinoma (SGC) is a rare type of cancer accounting for only 0.14% of all malignant neoplasms; it is estimated that 1.4 in 100,000 individuals are diagnosed with SGC per year^1^. According to the histological classification of salivary gland tumours by the World Health Organization, there are 20 histopathological types of SGC; their prognosis and biological characteristics considerably vary with the histological type^2^. Resection is the standard treatment for SGC regardless of the histopathological type and postoperative radiotherapy is recommended for patients at a high risk of recurrence^3^. Numerous clinical trials of cytotoxic chemotherapies have been conducted in patients with recurrent and/or metastatic (R/M) SGC^3,4^ and several potential targets for systemic therapy have been reported^5–17^; however, there were no randomised controlled trials^3,4^. Moreover, unlike lung metastasis of adenoid cystic carcinoma (AdCC), majority of which show indolent growth^3,18,19^, the progression of salivary duct carcinoma (SDC) and adenocarcinoma, not otherwise specified (NOS) is aggressive^3,20,21^. Hence, there is a need for systemic therapeutic strategies based on the histological characteristics for SGC.

Immune-checkpoint inhibitors (ICIs) have demonstrated durable antitumor effects against multiple cancer types, including head and neck squamous cell carcinoma^22,23^. Two prospective studies on pembrolizumab monotherapy^24^ and pembrolizumab combined with vorinostat^25^ for SGC have been published. Furthermore, two studies on nivolumab in patients with non-HNSCC, including six^26^ and two^27^ patients with SGC, respectively, have been published. However, these studies were not conducted exclusively in patients with SGC; to the best of our knowledge, there are no studies on the efficacy of nivolumab for SGC.

As a significant number of patients fail to benefit from ICIs; studies have been conducted to identify biomarkers to predict the response of patients to ICIs. Programmed death-ligand 1 (PD-L1) expression^22,23,28,29^ and microsatellite instability (MSI)^28,30^ are used as companion diagnostic markers; tumour mutation burden (TMB) is also one of the potential biomarkers of ICI response^28,29,31^. While most SGCs are microsatellite stable^32–34^, there are no consistent data on PD-L1 expression in SGCs due to the use of different antibodies and evaluation methods among studies^35–39^. Several case series studies of PD-L1 expression in SGCs including multiple histopathological types have showed a significantly higher positivity of PD-L1 in SDC than in other histopathological types of SGC^35–39^. Moreover, SDC is reported to harbour a higher mutational burden than other types of SGC^32,34,39–41^. Overall, these findings suggest that ICIs may have a higher efficacy against SDCs than other histopathological types of SGCs.

Recently, inflammatory markers including neutrophil-to-lymphocyte ratio (NLR), platelet-to-lymphocyte ratio (PLR), lymphocyte-to-monocyte ratio (LMR), serum C-reactive protein (CRP), modified Glasgow prognostic score (mGPS), absolute eosinophil count, and serum lactate dehydrogenase (LDH) have been reported to be useful predictors of progression of cancer including SDC^42,43^. Although it is largely unknown how systemic inflammation affects the survival of patients with cancer, neutrophils are known to promote tumour growth and distant metastasis by releasing cytokines that promote neovascularisation. As the number of lymphocytes reflects antitumor immunity, increased NLR due to neutrophilia and lymphocytopenia is associated with worse prognosis in patients treated with ICIs^44–46^. The LMR, PLR, CRP level, mGPS, absolute eosinophil count, and LDH have also been reported to correlate with the therapeutic effects of ICIs in patients with melanoma, non-small-cell lung cancer, and head and neck squamous cell carcinoma^46–51^. Therefore, in this retrospective multicentre study, we aimed to evaluate the efficacy of nivolumab monotherapy in patients with SGC including SDC. Additionally, we conducted a database analysis to determine the correlation between clinical profiles including PD-L1 positivity, MSI, and inflammatory biomarkers and the survival of patients with SGC treated with nivolumab.

## Results

### Patient characteristics and treatment

Twenty-four patients, identified in the cancer registry of the participating institutions during the study period, were enrolled in this study (Table 1). Written informed consent was obtained from all participants. The median follow-up period for all patients was 6.5 (range 0.6–28.2) months. All patients had received systemic therapy before nivolumab. The most common histopathological type of cancer was SDC (n = 20, 83%). Eleven tumours (46%) presented PD-L1 expression at a rate of ≥ 1%. Among them, three (13%) presented ≥ 50% PD-L1 positivity. Among 23 evaluable patients, none was classified as MSI-H.

**Table 1:** Baseline patients’ characteristics.

	n (%)
**Age (years)**	
Median (range)	56 (29–82)
**Sex**	
Male	19 (79)
Female	5 (21)
**Primary site**	
Parotid gland	19 (79)
Submandibular gland	3 (13)
Minor salivary gland	1 (4)
Accessory parotid gland	1 (4)
**Histopathology**	
Salivary duct carcinoma	20 (83)
Adenocarcinoma, NOS	2 (8)
Adenoid cystic carcinoma	1 (4)
Mucoepidermoid carcinoma	1 (4)
**Immunohistochemistry**	
HER2-positive^a^	11 (46)
AR-positive^b^	20 (83)
PD-L1 (28–8) < 1%	13 (54)
PD-L1 (28–8) 1–9%	5 (21)
PD-L1 (28–8) 10–49%	3 (13)
PD-L1 (28–8) ≥ 50%	3 (13)
MSI-H (n = 23)	0 (0)
**Prior treatment**	
None	0 (0)
Surgery	18 (75)
Radiotherapy	19 (79)
Concomitant radiotherapy (cisplatin)	9 (38)
Concomitant radiotherapy (carboplatin)	2 (8)
Systemic therapy	24 (100)
Systemic therapy for RM disease	22 (100)
Leuprorelin + bicalutamide	10 (42)
Carboplatin + paclitaxel	7 (29)
Trastuzumab + docetaxel	7 (29)
Carboplatin + docetaxel	5 (21)
S-1	5 (21)
Trastuzumab + S-1	5 (21)
Others^c^	13 (54)
Systemic therapy after nivolumab	6 (25)
Cetuximab + paclitaxel	5 (21)
Trastuzumab + docetaxel	1 (4)
Carboplatin + docetaxel	1 (4)
Abiraterone	1 (4)
S-1	1 (4)
Platinum refractory	12 (50)
**Target lesion**	
Locoregional	4 (17)
Locoregional + distant metastasis	1 (4)
Distant metastasis only	19 (79)
**Site of metastasis**	
Lung	10 (42)
Liver	5 (21)
Lymph nodes	4 (17)
Soft tissue (skin, muscle)	3 (13)
Bone	1 (4)
Brain, meninges	1 (4)
Pleura	1 (4)
Pericardium	1 (4)

*AR* androgen receptor, *CAB* combined androgen blockade, *HER2* human epidermal growth factor receptor 2, *mGPS* modified Glasgow prognostic score, *MSI-H* high-frequency microsatellite instability, *PD-L1* programmed death-ligand 1, *RM* recurrent/metastatic, *SDC* salivary duct carcinoma, *TPF* docetaxel/cisplatin/5-fluorouracil.

^a^The HER2 status was defined according to the American Society of Clinical Oncology/College of American Pathologists (ASCO/CAP) guidelines for breast cancer^54^^.^

^b^A case was considered to be AR-positive when ≥ 20% of the tumour cell nuclei showed strong staining^55^^.^

^c^Abiraterone, 3; bicalutamide, 2; enzalutamide, 2; docetaxel, cisplatin + docetaxel, carboplatin + pemetrexed, cisplatin + 5-fluorouracil, cisplatin + 5-fluorouracil + cetuximab and trastuzumab + docetaxel + pertuzumab, 1 each.

The median number of cycles of nivolumab administered was 8 (range 1–57). As of the cut-off date, 30 January 2020, two patients (8%) continued to receive nivolumab for 28 and 6 months, whereas 22 patients (92%) discontinued treatment due to PD (n = 19, 79%) and AEs (n = 3, 13%). Six patients (25%) received one or more of the following systemic therapy regimens after nivolumab treatment: cetuximab plus paclitaxel (n = 5, 21%), carboplatin plus docetaxel, trastuzumab plus docetaxel, abiraterone, and S-1 (n = 1, 4%, respectively).

### Response and survival outcomes

**Figure 1:**
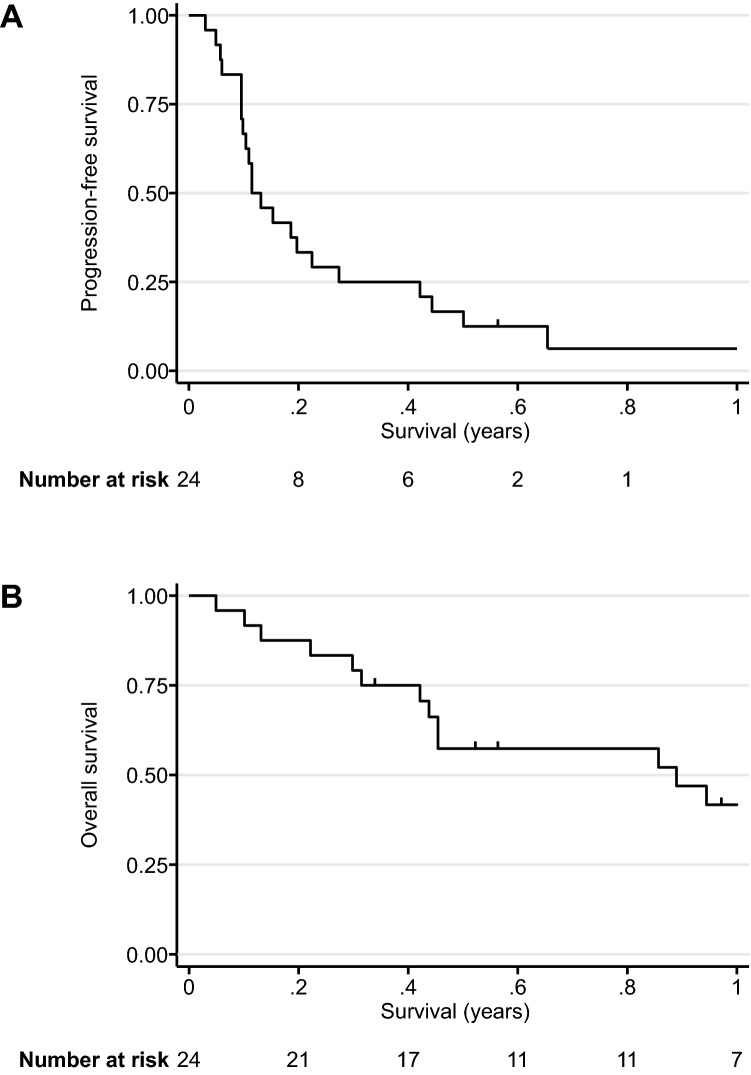
Kaplan–Meier curves of progression-free and overall survival. Kaplan–Meier curves of (**A**) progression-free survival and (**B**) overall survival. The vertical lines indicate censored events.

**Figure 2:**
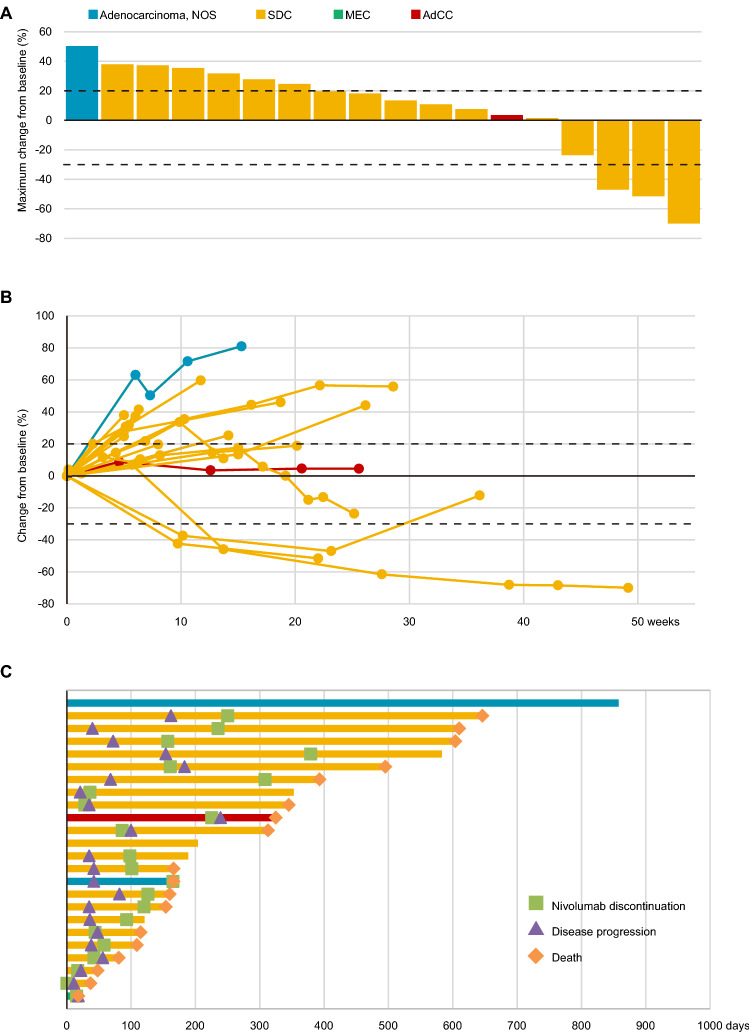
Characteristics of responses in patients with salivary gland carcinoma treated with nivolumab according to Response Evaluation Criteria in Solid Tumours (version 1.1) based on histopathological diagnosis. (**A**) The highest reduction from the baseline in target lesions. Tumour shrinkage relative to the baseline was observed in four patients (16.7%). The upper dotted lines represent the threshold for progressive disease (a 20% increase in the sum of the longest diameter of the target lesions) and the lower dotted lines represent the threshold for a partial response (a 30% decrease in the sum of the longest diameter of the target lesions). (**B**) Change from the baseline (%) in the sum of the target lesions over time to progressive disease. The upper dotted lines represent the threshold for progressive disease (a 20% increase in the sum of the longest diameter of the target lesions) and the lower dotted lines show the threshold for a partial response (a 30% decrease in the sum of the longest diameter of the target lesions). (**C**) Time to response and the duration of survival. Each bar represents an individual patient, with the length of the bar corresponding to the time of overall survival based on the disease status. *SDC* salivary duct carcinoma, *MEC* mucoepidermoid carcinoma, *AdCC* adenoid cystic carcinoma.

**Figure 3:**
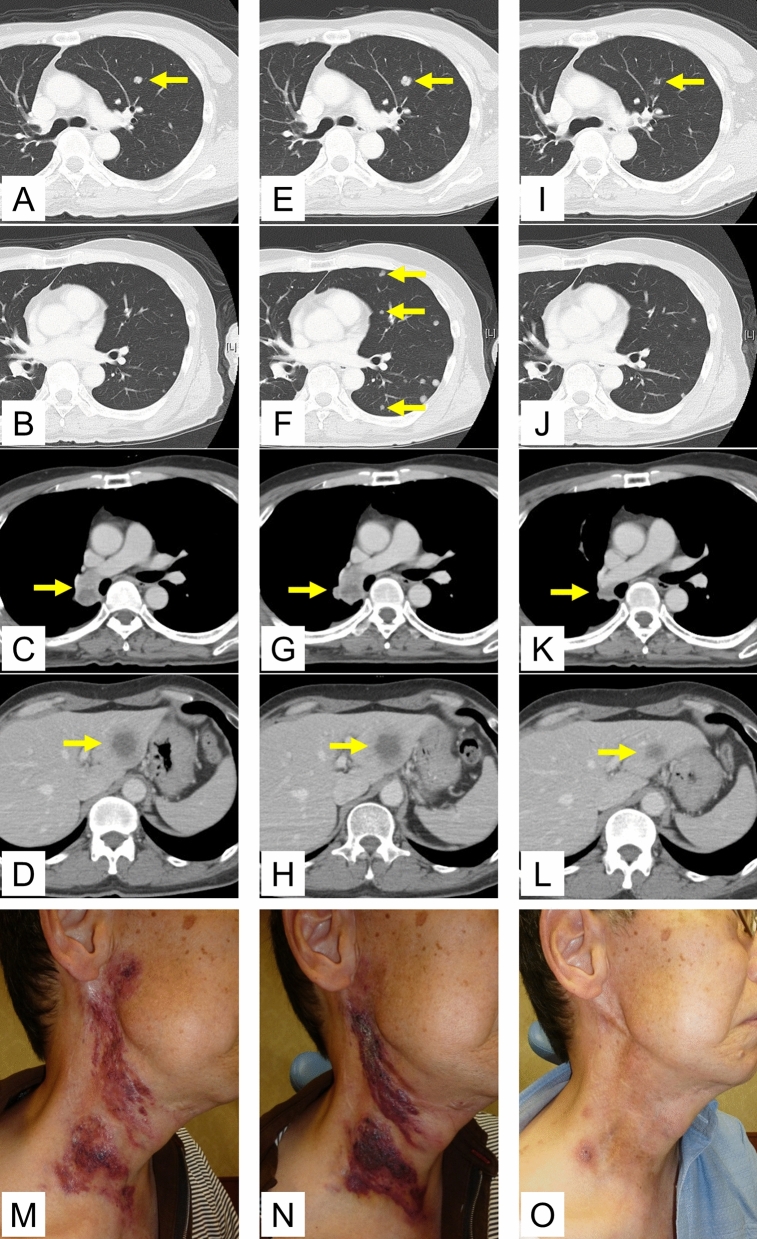
Representative images of the tumor before and during nivolumab monotherapy in two patients with recurrent and/or metastatic salivary gland carcinoma. (**A**–**D**) Pre-treatment of a patient with lung, liver, and hilar lymph node metastasis, (**E**–**H**) 40 days after the initiation of nivolumab treatment, (**I**–**L**) 96 days after the initiation of nivolumab treatment. Tumor shrinkage was observed in this patient following PD diagnosis due to a new lesion. The yellow arrows indicate lung metastases (**A**, **E**, **I**), new lung lesion (**F**), hilar lymph node metastasis (**C**, **G**, **K**), and liver metastases (**D**, **H**, **L**). (**M**–**O**) Pre-treatment of a patient with cervical skin metastasis, (**P**–**R**) 57 days after the initiation of nivolumab treatment, (**S**–**U**) 204 days after the initiation of nivolumab treatment. Tumor shrinkage was observed in this patient after an increase in skin tumor thickness, which was in the SD range.

The therapeutic efficacy of nivolumab are shown in Table 2. None of the patients achieved CR; 1 (4%), 2 (8%), and 21 (88%) patients showed PR, SD, and PD, respectively. The ORR was 4.2% (95% CI 0.1–21.1%). Two patients with SD maintained the status for more than 24 weeks. Thus, both CBR and DCR were 12.5% (95% CI 2.7–32.4%). The Kaplan–Meier survival curves of PFS and OS of all patients are shown in Fig. 1; the median PFS was 1.6 (95% CI 1.2–4.4) months and the median OS was 10.7 (95% CI 5.1–19.8) months. The therapeutic effects observed in 20 patients with SDC were as follows: ORR, 5.0% (95% CI 2.7–24.9%); median PFS, 1.5 (95% CI 1.1–2.7) months; and median OS, 11.3 (95% CI 3.8–19.8) months. Figure 2 shows the waterfall, spider, and swimmer plots of all patients based on the histopathological diagnosis. Figure 3 shows the representative images of tumour before and during nivolumab monotherapy in two patients.

**Table 2:** Treatment efficacy.

Efficacy	All patients (n = 24)	Salivary duct carcinoma (n = 20)
Complete response, n (%)	0 (0)	0 (0)
Partial response, n (%)	1 (4.2)	1 (5.0)
Stable disease, n (%)	2 (8.3)	0 (0)
Progressive disease, n (%)	21 (87.5)	19 (95.0)
Objective response^a^, n (%, 95% CI)	1 (4.2, 0.1–21.1)	1 (5.0, 2.7–24.9)
Disease control^b^, n (%, 95% CI)	3 (12.5, 2.7–32.4)	1 (5.0, 2.7–24.9)
Stable disease ≥ 24 weeks, n (%)	2 (8.3)	0 (25.0)
Clinical benefit^c^, n (%, 95% CI)	3 (12.5, 2.7–32.4)	1 (5.0, 2.7–24.9)
Median progression-free survival, months (95% CI)	1.6 (1.2–4.4)	1.5 (1.1–2.7)
Median overall survival, months (95% CI)	10.7 (5.1–19.8)	11.3 (3.8–19.8)

*CI* confidence interval, *NR* not reached.

^a^Confirmed complete and partial responses.

^b^Complete response, partial response, and stable disease.

^c^Complete response, partial response, and stable disease ≥ 24 weeks.

### Safety

All AEs reported are listed in Table 3. Twenty-two patients (92%) experienced at least one AE during the treatment. Six patients (25%) showed grade 3 or 4 AEs; 1 patient showed grade 4 increase in creatine phosphokinase (4%); 3 patients (13%) showed grade 3 anaemia, and 1 patient each showed an increase in alkaline phosphatase, amylase, aspartate aminotransferase, alanine transaminase, hyponatraemia, and myositis (4%). No treatment-related death was observed. Frequent AEs of all grades included anaemia (n = 17, 71%), increased alkaline phosphatase (n = 10, 42%), and hypoalbuminemia (n = 9, 38%). Five patients (21%) had an irAE, and only one of these patients (4%) had grade 4 increase in creatine phosphokinase and grade 3 myositis. Other irAEs reported were grade 2 pneumonitis (n = 1, 4%), grade 1 hyperthyroidism (n = 2, 8%), and hypothyroidism (n = 1, 4%). Three patients discontinued treatment due to AEs including grade 3 myositis (n = 1) and grade 2 pneumonitis (n = 2).

**Table 3:** Reported adverse events, n (%).

Event	Any grade	Grade 1	Grade 2	Grade 3	Grade 4
Any	22 (92)	19 (79)	8 (33)	6 (25)	1 (4)
Anaemia	17 (71)	11 (46)	3 (13)	3 (13)	0
ALP increased	10 (42)	8 (33)	1 (4)	1 (4)	0
Hypoalbuminemia	9 (38)	7 (29)	2 (8)	0	0
Hyperkalaemia	6 (25)	3 (17)	3 (13)	0	0
Heart failure	6 (25)	6 (25)	0	0	0
Serum amylase increased	5 (21)	3 (13)	1 (4)	1 (4)	0
AST increased	5 (21)	4 (17)	0	1 (4)	0
Hyponatraemia	4 (17)	3 (13)	0	1 (4)	0
CPK increased	3 (13)	0	2 (8)	0	1 (4)
ALT increased	3 (13)	2 (8)	0	1 (4)	0
Cre increased	3 (13)	1 (4)	2 (8)	0	0
Chronic kidney disease	2 (8)	0	2 (8)	0	0
γ-GTP increased	2 (8)	1 (4)	1 (4)	0	0
Pneumonitis	2 (8)	0	2 (8)	0	0
Hyperthyroidism	2 (8)	2 (8)	0	0	0
Hypertriglyceridaemia	2 (8)	2 (8)	0	0	0
Hypokalaemia	2 (8)	2 (8)	0	0	0
APTT prolonged	2 (8)	2 (8)	0	0	0
Blood LDH increased	2 (8)	2 (8)	0	0	0
Hyperglycaemia	2 (8)	2 (8)	0	0	0
Myositis	1 (4)	0	0	1 (4)	0
Lymphocyte count decreased	1 (4)	0	1 (4)	0	0
INR increased	1 (4)	0	1 (4)	0	0
White blood cell decreased	1 (4)	1 (4)	0	0	0
Platelet count decreased	1 (4)	1 (4)	0	0	0
Hypothyroidism	1 (4)	1 (4)	0	0	0
Cholesterol high	1 (4)	1 (4)	0	0	0
Hyperuricaemia	1 (4)	1 (4)	0	0	0
Hypophosphataemia	1 (4)	1 (4)	0	0	0
Arthralgia	1 (4)	1 (4)	0	0	0

*ALP* alkaline phosphatase, *ALT* alanine aminotransferase, *APTT* activated partial thromboplastin time, *AST* aspartate aminotransferase, *CPK* creatinine phosphatase, *γ-GTP* γ-glutamyl transpeptidase, *LDH* lactate dehydrogenase, *INR* international normalised ratio.

### Exploratory analysis of biomarkers of ICI response

**Figure 4:**
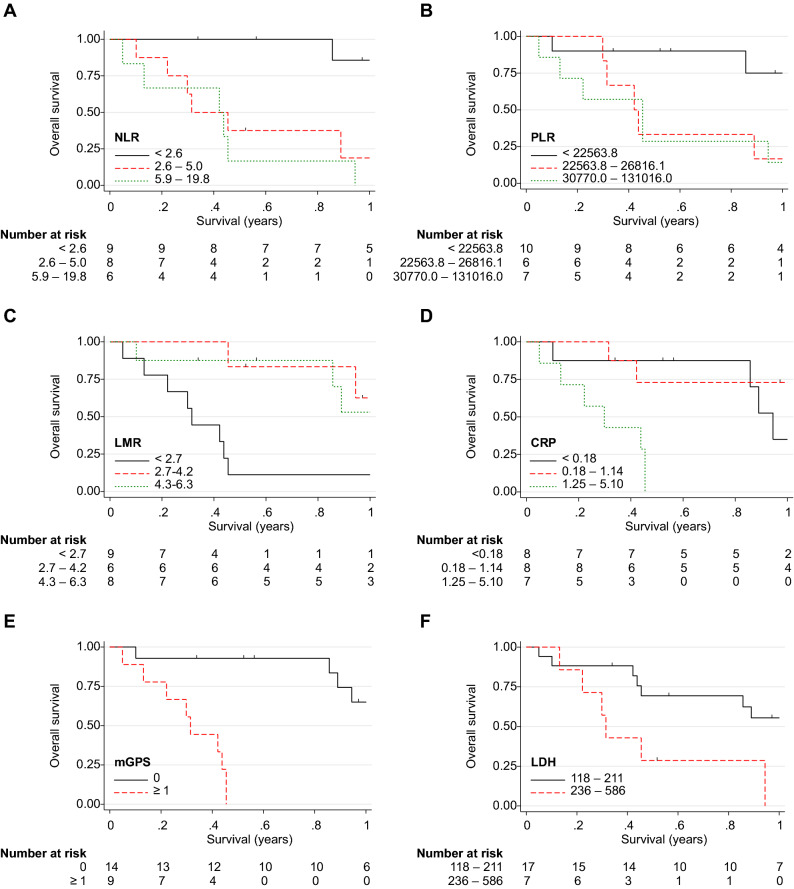
Kaplan–Meier curves of overall survival in patients with salivary gland carcinoma stratified by (**A**) the neutrophil-to-lymphocyte ratio (NLR), (**B**) platelet-to-lymphocyte ratio (PLR), (**C**) lymphocyte-to-monocyte ratio (LMR), (**D**) C-reactive protein (CRP), (**E**) modified Glasgow prognostic score (mGPS), and (**F**) lactate dehydrogenase (LDH) activity.

Table 4 and Fig. 4 show the results of the exploratory analysis of the biomarkers. As all patients were microsatellite stable, no analysis was performed according to the MSI status. There was no association between PD-L1 positivity and prognosis. In the univariate analysis, higher NLR, higher serum LDH and CRP, and lower LMR were significantly associated with shorter PFS. The significant predictors of a shorter OS were the ECOG PS ≥ 1, mGPS ≥ 1, higher NLR, higher serum LDH, and higher serum CRP. Systemic therapy following nivolumab and higher LMR were significantly associated with a longer OS. The Kaplan–Meier curves of the OS and PFS, waterfall, spider, and swimmer plots according to the biomarkers are presented in Fig. 3, Supplementary Figs. S1 and S2, respectively. The 1-year OS of patients with the ECOG PS 0, with systemic therapy after nivolumab, mGPS 0, lower NLR, higher LMR, lower LDH level, and lower CRP level was 59.3%, 83.3%, 65.0%, 85.7%, 52.5%, 55.5%, and 68.6%, respectively (Fig. 3).

**Table 4:** Exploratory analysis of the biomarkers.

	N	Progression-free survival			Overall survival		
		HR	95% CI	*P*-value	HR	95% CI	*P*-value
**Age**							
< 65 years	14	1.00	–	–	1.00	–	–
≥ 65 years	10	1.81	0.76–4.30	0.181	1.05	0.37–2.96	0.931
**Sex**							
Male	19	1.00	–	–	1.00	–	
Female	5	0.53	0.15–1.85	0.322	1.27	0.40–4.00	0.686
**ECOG PS**							
0	13	1.00	–	–	1.00	–	–
≥ 1	11	1.99	0.85–4.68	0.115	2.87	1.08–7.61	0.034
**Primary site**							
Parotid gland	19	1.00	–	–	1.00	–	–
Others	5	0.62	0.21–1.86	0.393	1.04	0.33–3.25	0.945
**Prior systemic therapy**							
−	2	1.00	–	–	1.00	–	–
+	22	2.95	0.37–23.28	0.305	2.72	0.33–22.11	0.350
**irAE**							
−	19	1.00	–	–	1.00	–	–
+	5	1.99	0.70–5.64	0.196	2.27	0.69–7.47	0.178
**Systemic therapy after nivolumab**							
−	15	1.00	–	–	1.00	–	–
+	7	0.82	0.32–2.08	0.673	0.06	0.01–0.48	0.008
**Histopathology**							
Salivary duct carcinoma	20	1.00	–	–	1.00	–	–
Others	4	0.36	0.08–1.58	0.176	0.81	0.23–2.88	0.746
**PD-L1**							
0%	13	1.00	–	–	1.00	–	–
≥ 1%	11	0.85	0.36–2.03	0.716	0.73	0.25–2.10	0.558
**mGPS**							
0	14	1.00	–	–	1.00	–	–
≥ 1	9	2.69	0.99–7.30	0.052	30.06	3.66–246.95	0.002
**Neutrocyte-to-lymphocyte ratio**							
< 2.6	9	1.00	–	–	1.00	–	–
2.6–5.0	8	2.04	0.72–5.79	0.179	8.90	1.81–43.80	0.007
5.9–19.8	6	4.02	1.24–13.07	0.021	15.48	2.82–85.04	0.002
		P_trend_ = 0.023			P_trend_ = 0.001		
**Platelet-to-lymphocyte ratio**							
< 22,563.8	10	1.00		–	1.00		–
22,563.8–26,816.1	6	1.50	0.51–4.41	0.463	4.69	1.16–18.97	0.030
30,770–131,016	7	2.94	0.95–9.09	0.061	5.67	1.43–22.38	0.013
		P_trend_ = 0.076			P_trend_ = 0.011		
**Lymphocyte-to-monocyte ratio**							
< 2.7	9	1.00		–	1.00		–
2.7–4.2	6	1.44	0.49–4.18	0.507	0.24	0.07–0.84	0.025
4.3–6.3	8	0.11	0.02–0.57	0.008	0.14	0.04–0.56	0.006
		P_trend_ = 0.015			P_trend_ = 0.005		
**LDH**							
118–211	17	1.00	–	–	1.00	–	–
236–586	7	3.09	1.06–8.98	0.039	3.42	1.11–10.50	0.032
**CRP**							
< 0.18	8	1.00	–	–	1.00	–	–
0.18–1.14	8	3.92	1.08–14.23	0.038	1.18	0.31–4.48	0.810
1.25–5.10	7	5.08	1.29–20.05	0.020	10.58	2.25–49.89	0.003
		P_trend_ = 0.028			P_trend_ = 0.007		

*HR* hazard ratio, *CI* confidence interval, *irAE* immune-related adverse event, *PD-L1* programmed death-ligand 1, *mGPS* modified Glasgow prognostic score, *LDH* lactate dehydrogenase, *CRP* C-reactive protein.

## Discussion

In the present retrospective study of nivolumab monotherapy in 24 patients with R/M SGC, the ORR was 4.2%, with the median PFS and OS of 1.6 and 10.7 months, respectively. The ORR of the 20 patients with SDC was 5.0% and the median PFS and OS were 1.5 and 11.3 months, respectively. Nivolumab was well tolerated by patients with SGC, and AEs associated with nivolumab was comparable with those associated with pembrolizumab^24^. In this study, the therapeutic effects were limited; however, some patients achieved considerably long-term disease control.

Prospective studies on pembrolizumab^24^ and pembrolizumab combined with vorinostat^25^ reported that the ORR of patients with multiple histopathological types of SGC was 12% and 16%, respectively, with the median PFS and OS of 4–6.9 and 13–14 months, respectively. The ORR and the median OS of patients with SGC to nivolumab in this study were comparable with those observed in patients with advanced SGC treated with pembrolizumab^24,25^. However, the median PFS of this study was shorter than that reported by the previous study on pembrolizumab. This could be because only patients with obvious progression within 6 months were included in this study, whereas the previous studies with pembrolizumab did not adopt this criterion.

In the present study, the results of the biomarker analysis revealed that most factors related to PS and inflammatory biomarkers such as the NLR, LMR, LDH, and CRP levels were associated with the prognosis of tumour in patients treated with nivolumab. To the best of our knowledge, this is the first study to demonstrate an association between inflammatory biomarkers and prognosis in patients with SGC treated with nivolumab. In particular, NLR showed an apparent negative dose–response relationship with the OS; the 1-year OS of patients with lower NLR was 85.7%. The NLR, LMR, PLR, CRP level, mGPS, and LDH level have also been reported to correlate with the therapeutic effects of ICIs in various cancers^46–51^. As a result, NLR, LMR, PLR, LDH, CRP, and mGPS are suggested to reflect the general conditions (immunological competence) of the host, and they can be used as biomarkers of ICI treatment response.

In malignant melanoma, non-small cell lung cancer, and head and neck squamous cell carcinoma, PD-L1 immunohistochemistry^22,23,28,29^ including the presence of PD-L1-expressing immune cells in the tumour microenvironment^52,53^, mismatch repair (MMR)/MSI^29,31^, and TMB^29–31^ have been reported as biomarkers of ICI response. Based on the findings of previous studies, which showed significantly higher PD-L1 expression and TMB in SDC than those in other tumours^32–41^, higher therapeutic effects of ICIs are being expected. However, the efficacy of ICI monotherapy for SDC was limited in our cohort. This might be since no tumour was MSI-H and the PD-L1-positivity rate in the tumour cells was low in our SDC cohort.

While previous studies on anti-HER2 antibody including trastuzumab^5–8^ and androgen deprivation therapy (e.g., bicalutamide and leuprorelin)^9–12^ for HER2- or AR-positive patients with SDC showed the ORR was 20–89%, the response rate of patients to nivolumab in this study was unsatisfactory. Thus, nivolumab monotherapy is not recommended for patients with HER2- or AR-positive advanced SDCs before anti-HER2- or AR-targeted therapy. In contrast, ICIs might be tried in patients with SGC without targetable molecules instead of conventional cytotoxic anticancer agents. Recent studies reported that cytotoxic anticancer agents seemed to achieve a higher ORR with higher toxicity than the ICIs^3,4,24,25^. However, it is difficult to directly compare those therapies as patient background (e.g., histological type) might differ. As our data suggest low nivolumab ORR and shorter survival in patients with increased systemic inflammatory markers (e.g., NLR), the use of cytotoxic anticancer agents may be prioritised in symptomatic patients (e.g., patients with pain and/or aggressive tumour growth) and patients with increased systemic inflammatory markers. Currently, a clinical trial on the combination of pembrolizumab and docetaxel in patients with thyroid cancer or SGC without standard-of-care treatment is under progress (ClinicalTrials.gov Identifier: NCT03360890). Other ongoing clinical trials targeting patients with SGC include the combination of pembrolizumab and lenvatinib (ClinicalTrials.gov Identifier: NCT04209660), two ICIs (nivolumab and ipilimumab; ClinicalTrials.gov Identifier: NCT02834013, NCT03146650 and NCT03172624), and ICIs and AR-targeted therapy (pembrolizumab and goserelin acetate; ClinicalTrials.gov Identifier: NCT03942653).

This study had some limitations. First, owing to the retrospective nature and small sample size of the study, the superiority of nivolumab over other drugs was not examined. Second, the biomarkers identified in this study including NLR might be merely prognostic factors, which are associated with survival and might not predict response to nivolumab. Moreover, the optimal cut-off value for NLR was unknown. Thus, future clinical trials with a larger sample size should be performed to address these issues.

In the present study, the efficacy of nivolumab monotherapy for SGC was limited. However, some patients achieved long-term disease control with nivolumab. Further studies are warranted to elucidate a predictive factor of ICI in patients with advanced SGC.

## Materials and methods

### Patients and treatment

This was a multicentre retrospective cohort study conducted in Japan. Following approval from the ethics committee of the participating institutions (Approval number of each institution: International University of Health and Welfare, Mita Hospital, 5-18-50; Nihonkai General Hospital, 30-(4)-3; Niigata University, 2019-0056; Tokyo Medical University, T2018-0059; Nagoya City University, 60-20-0049), data of patients with unresectable R/M SGC treated with nivolumab between May 2017 and September 2019 were extracted from the database of the nation-wide cancer registry of each participating institution. This study was conducted in accordance with the Declaration of Helsinki. Written informed consent was obtained from each patient and/or their legal guardians. Additionally, we obtained informed consent of the patients for publication of identifying images and photographs.

Patients with ≥ 20% tumour growth within 6 months prior to treatment detected by computed tomography (CT) scan, magnetic resonance imaging, and/or positron emission tomography-CT were treated with nivolumab (240 mg) every 2 weeks. The treatment dose and duration were determined in accordance with the Japanese guidelines for head and neck cancer, including salivary gland cancer. A pathological review of all patients was performed by a pathologist with expertise in SGCs (T.N.). Carcinoma ex pleomorphic adenomas were classified into different histopathological types according to each carcinoma component instead of a separate category. Imaging tests were performed every 6–8 weeks.

### Immunohistochemical and gene alteration analyses

The expression status of PD-L1, MSI, HER2, and androgen receptor (AR) in patients administered nivolumab was also obtained from the database of the participating institutes. The expression level of PD-L1 in the resected or biopsy specimens of tumours was analysed using the rabbit antihuman PD-L1 clone 28-8 using the automated immunohistochemical assay (PD-L1 IHC 28-8 pharmDx; Dako-Agilent Technologies, Santa Clara, CA, USA). PD-L1 expression was defined as the percentage (instead of intensity) of tumour cells exhibiting plasma membrane staining^22^.

The MSI test kit (product code: 4987931010017; FALCO Biosystems, Kyoto, Japan) was used to evaluate MSI as described previously^54^. Briefly, a polymerase chain reaction (PCR) of microsatellite markers at five loci (BAT25, BAT26, NR21, NR24, and MONO27) was conducted using DNA extracted from tumour specimens. In normal patients, the PCR products were in the quasi-monomorphic variation range (QVR). Specimens with the PCR products outside the QVR were classified as MSI-positive. Specimens with more than one positive locus were classified as MSI-high (MSI-H).

HER2 and AR statuses were assessed as described previously^5,10,55,56^. Briefly, specimens with 3 + HER2 immunoreactivity or *HER2* gene amplification were classified as HER2-positive according to the guidelines for breast cancer by the American Society of Clinical Oncology and the College of American Pathologists^56^. AR was classified as positive if ≥ 20% of nuclei in tumour cells were immunoreactive.

### Analysis of biomarkers of ICI response

An exploratory analysis of potential biomarkers of ICI response was performed^43,46^. The associations between prognosis and age, sex, the Eastern Cooperative Oncology Group (ECOG) performance status (PS), prior systemic therapy (present or absent), immune-related adverse events (irAE; present or absent), systemic therapy after nivolumab (present or absent), histopathological type (SDC or non-SDC), PD-L1 status, HER2 status, AR status, MSI status, mGPS, NLR, PLR, LMR, serum CRP, LDH, and absolute eosinophil count were examined.

### Statistical analysis

The therapeutic effect of nivolumab was evaluated according to the overall response rate (ORR), which was defined as the percentage of patients who achieved complete response (CR) or partial response (PR), clinical benefit rate [CBR, defined as the percentage of patients who achieved CR, PR, or stable disease (SD) for at least 24 weeks], disease control rate (DCR, defined as the percentage of patients who achieved CR, PR, or SD regardless of duration), median progression-free survival (PFS), and median overall survival (OS)^5,10,43,46^. Treatment efficacy was evaluated according to Response Evaluation Criteria in Solid Tumors version 1.1 (RECIST 1.1)^57^. PFS was defined as the time from the start of nivolumab treatment to the diagnosis of progressive disease (PD). OS was defined as the time from the start of nivolumab treatment to death from any cause. Safety was evaluated according to Common Terminology Criteria for Adverse Events (CTCAE) ver. 5.0^58^. The Kaplan–Meier method was used to estimate PFS and OS. The Cox proportional hazards model was used to calculate the hazard ratio (HR) with 95% confidence interval (CI). The results with a *P* value of < 0.05 were considered statistically significance. All analyses were performed using STATA ver. 16 (StataCorp, College Station, TX, USA).

## Supplementary information

Supplementary Information.

## Data Availability

The datasets generated in the current study are available from the corresponding author on request.
