# Supplementary material for: Multicentre, retrospective study of the efficacy and safety of nivolumab for recurrent and metastatic salivary gland carcinoma
Source: Sci Rep. 2020 Oct 12;10:16988. doi: 10.1038/s41598-020-73965-6 (PMC7552420; doi:10.1038/s41598-020-73965-6)
Supplement: Supplementary file 1 — Supplementary Information. [file 41598_2020_73965_MOESM1_ESM.pdf]

Multicentre, retrospective study of the efficacy and safety of nivolumab for recurrent and metastatic salivary gland carcinoma

Kazutomo Niwa[1,2,+], Daisuke Kawakita[3,+], Toshitaka Nagao[4], Hideaki Takahashi[2], Takashi Saotome[5], Masashi Okazaki[6], Keisuke Yamazaki[7], Isaku Okamoto[8], Hideaki Hirai[4], Natsuki Saigusa[4], Chihiro Fushimi[1], Tatsuo Masubuchi[1], Kouki Miura[1], Shin-ichi Okazaki[6], Hirooki Matsui[6], Takuro Okada[8], Sho Iwaki[3], Takashi Matsuki[9], Kenji Hanyu[1], Kiyoaki Tsukahara[8], Nobuhiko Oridate[2], and Yuichiro Tada[1,*,+]

[1]Department of Head and Neck Oncology and Surgery, International University of Health and Welfare, Mita Hospital, Tokyo, 108-8329, Japan
[2]Department of Otorhinolaryngology, Head and Neck Surgery, Yokohama City University, School of Medicine, Yokohama, Kanagawa, 236-0004, Japan
[3]Department of Otorhinolaryngology, Head and Neck Surgery, Nagoya City University Graduate School of Medical Sciences, Nagoya, 467-8602, Japan
[4]Department of Anatomic Pathology, Tokyo Medical University, Tokyo, 160-0023, Japan
[5]Division of Medical Oncology, Matsudo City Hospital, Chiba, 270-2252, Japan
[6]Department of Otorhinolaryngology, Head and Neck Surgery, Nihonkai General Hospital, Yamagata, 998-8501, Japan
[7]Department of Otolaryngology Head and Neck Surgery, Niigata University Graduate School of Medical and Dental Sciences, Niigata, 951-8520, Japan
[8]Department of Otorhinolaryngology Head and Neck Surgery, Tokyo Medical University, Tokyo, 160-0023, Japan
[9]Department of Otorhinolaryngology, Head and Neck Surgery, Kitasato University School of Medicine, Sagamihara, 252-0375, Japan

*Corresponding author: ytada@iuhw.ac.jp
+These authors contributed equally to this work

Supplementary Data

Supplementary Figure S1

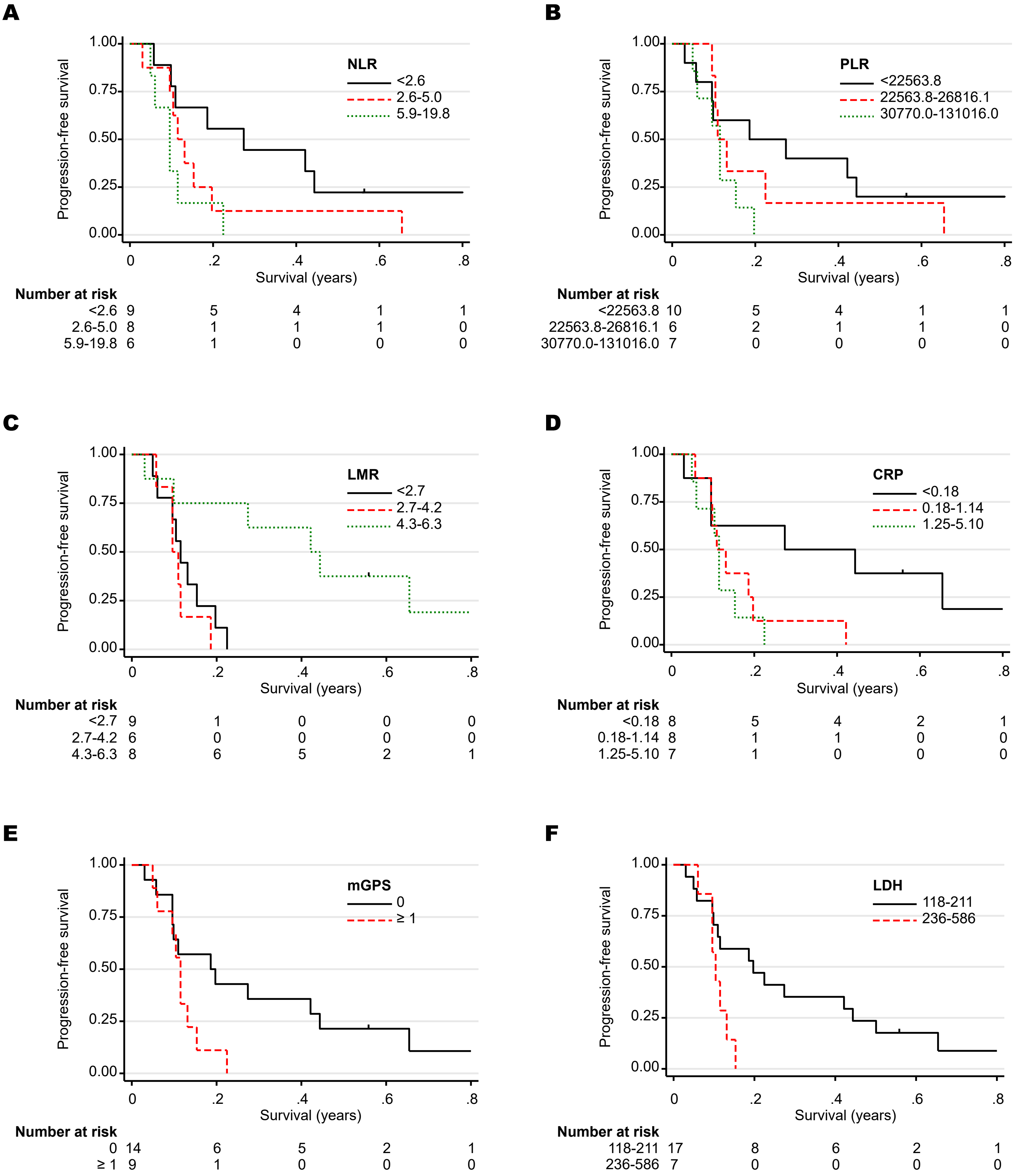

Supplementary Figure S1.

Kaplan-Meier curves of progression-free survival in patients with salivary gland carcinoma stratified by the neutrophil-to-lymphocyte ratio (NLR; A), platelet-to-lymphocyte ratio (PLR; B), lymphocyte-to-monocyte ratio (LMR; C), C-reactive protein (CRP; D) level, modified Glasgow prognostic score (mGPS; E), and lactate dehydrogenase (LDH; F).

Supplementary Figure S2.

Waterfall, spider, and swimmer plots of the patients with salivary gland carcinoma treated with nivolumab monotherapy stratified by the expression of programmed death-ligand 1 (PD-L1; A-C), neutrophil-to-lymphocyte ratio (NLR; D-F), platelet-to-lymphocyte ratio (PLR; G-I), lymphocyte-to-monocyte ratio (LMR; J-L), C-reactive protein level (CRP; M-O), modified Glasgow prognostic score (mGPS; P-R), and lactate dehydrogenase (LDH; S-U), respectively.

Supplementary Figure S2

A

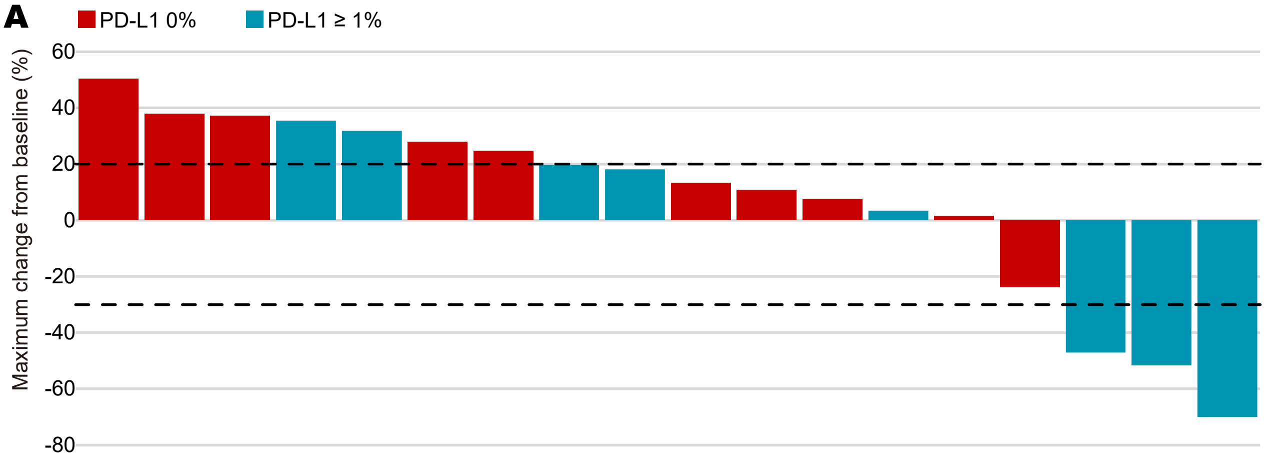

B

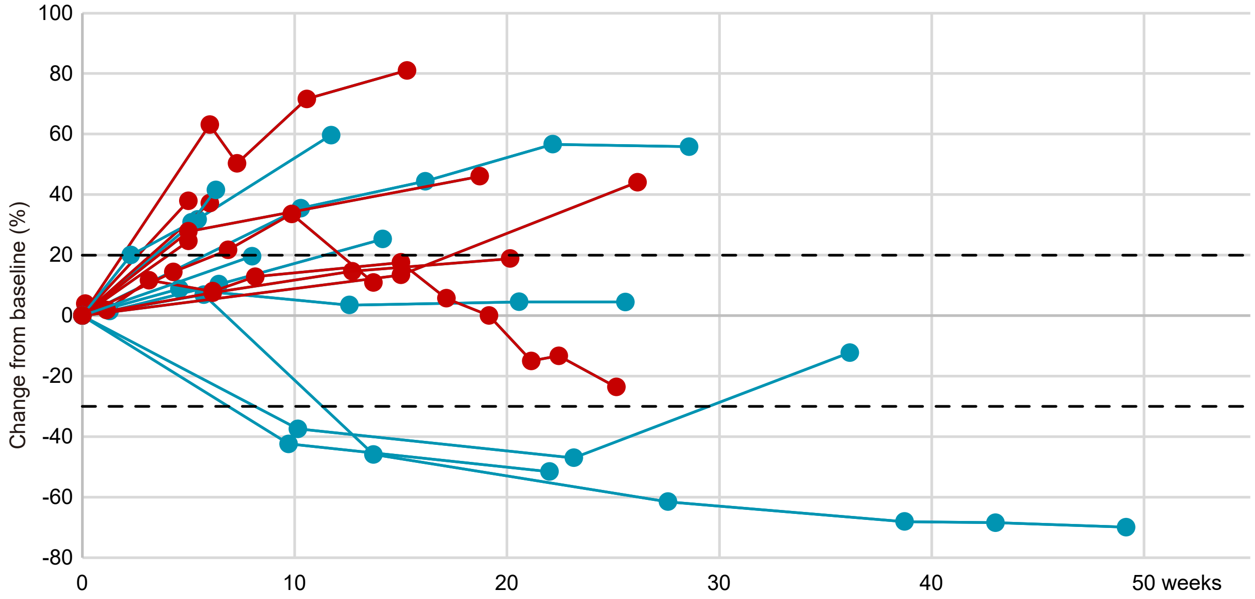

C

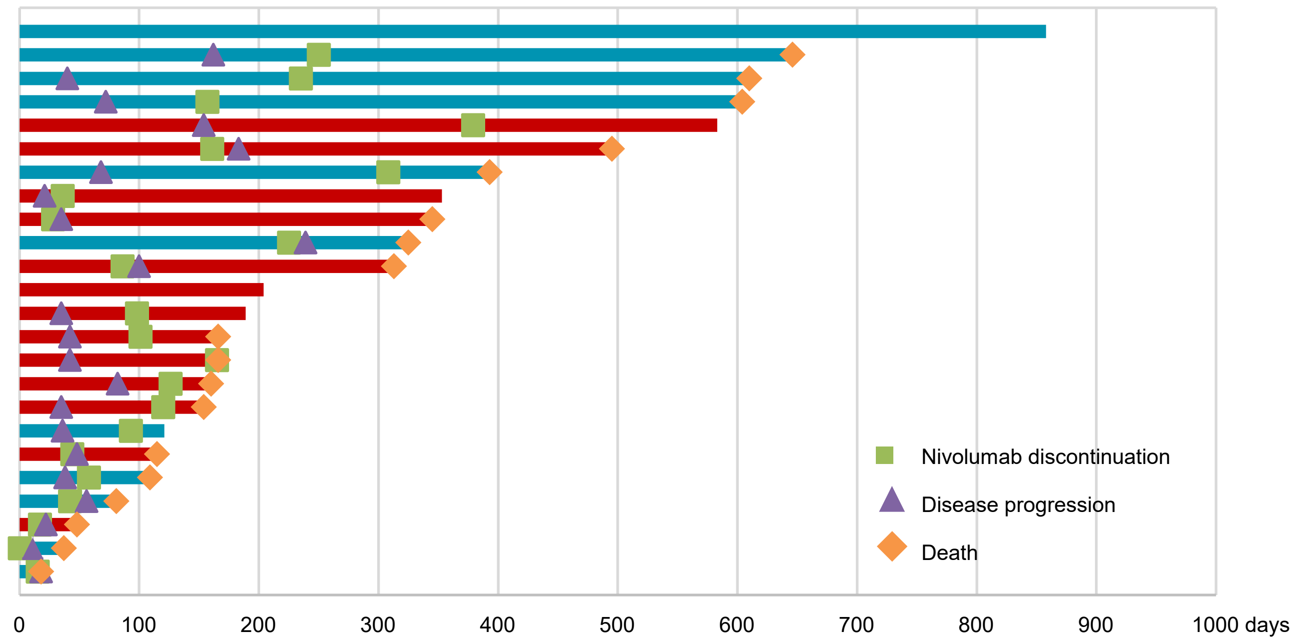

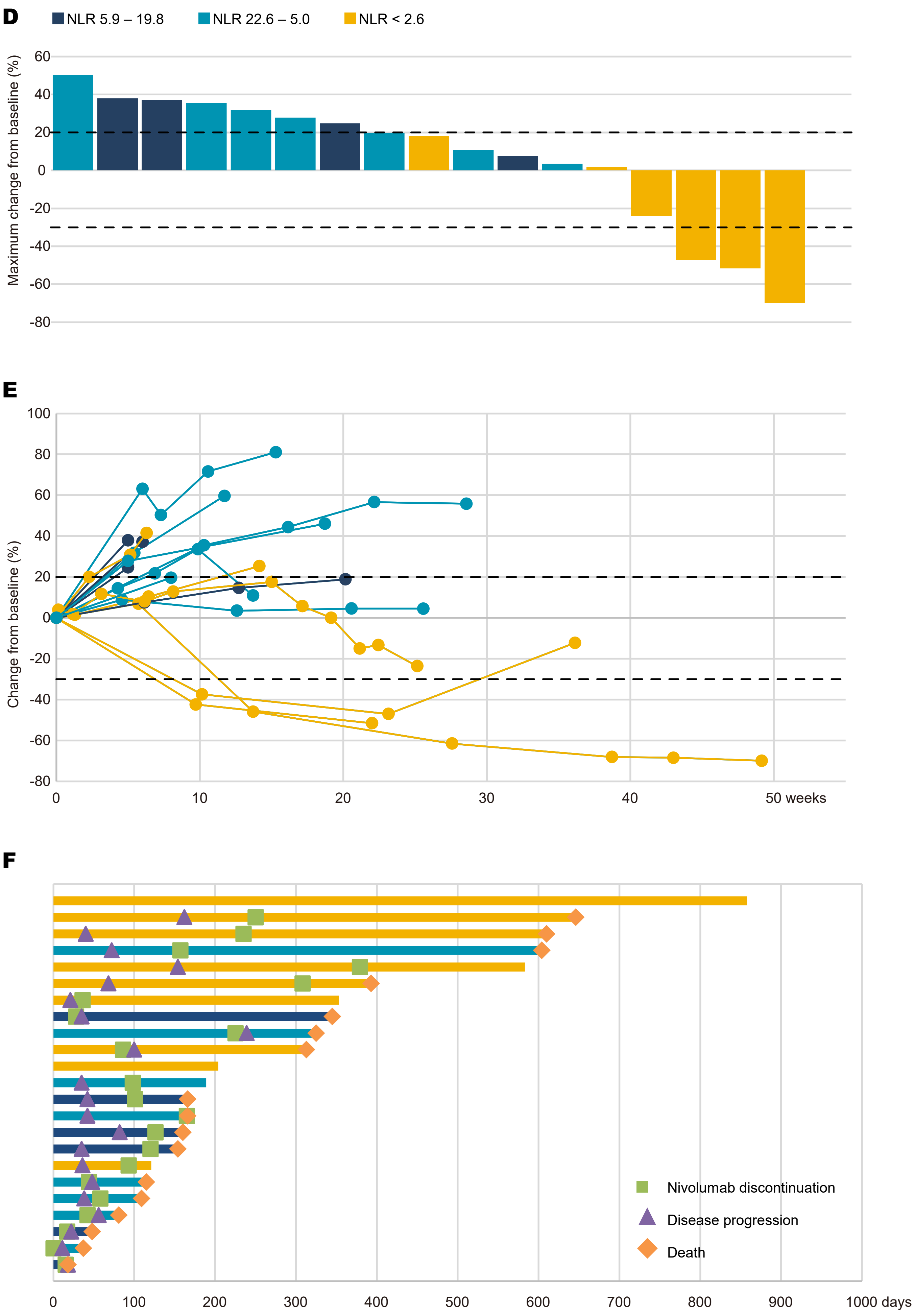
D
NLR 5.9 – 19.8
NLR 22.6 – 5.0
NLR < 2.6
Maximum change from baseline (%)
60
40
20
0
-20
-40
-60
-80
E
Change from baseline (%)
100
80
60
40
20
0
-20
-40
-60
-80
0
10
20
30
40
50 weeks
F
Nivolumab discontinuation
Disease progression
Death
0
100
200
300
400
500
600
700
800
900
1000 days

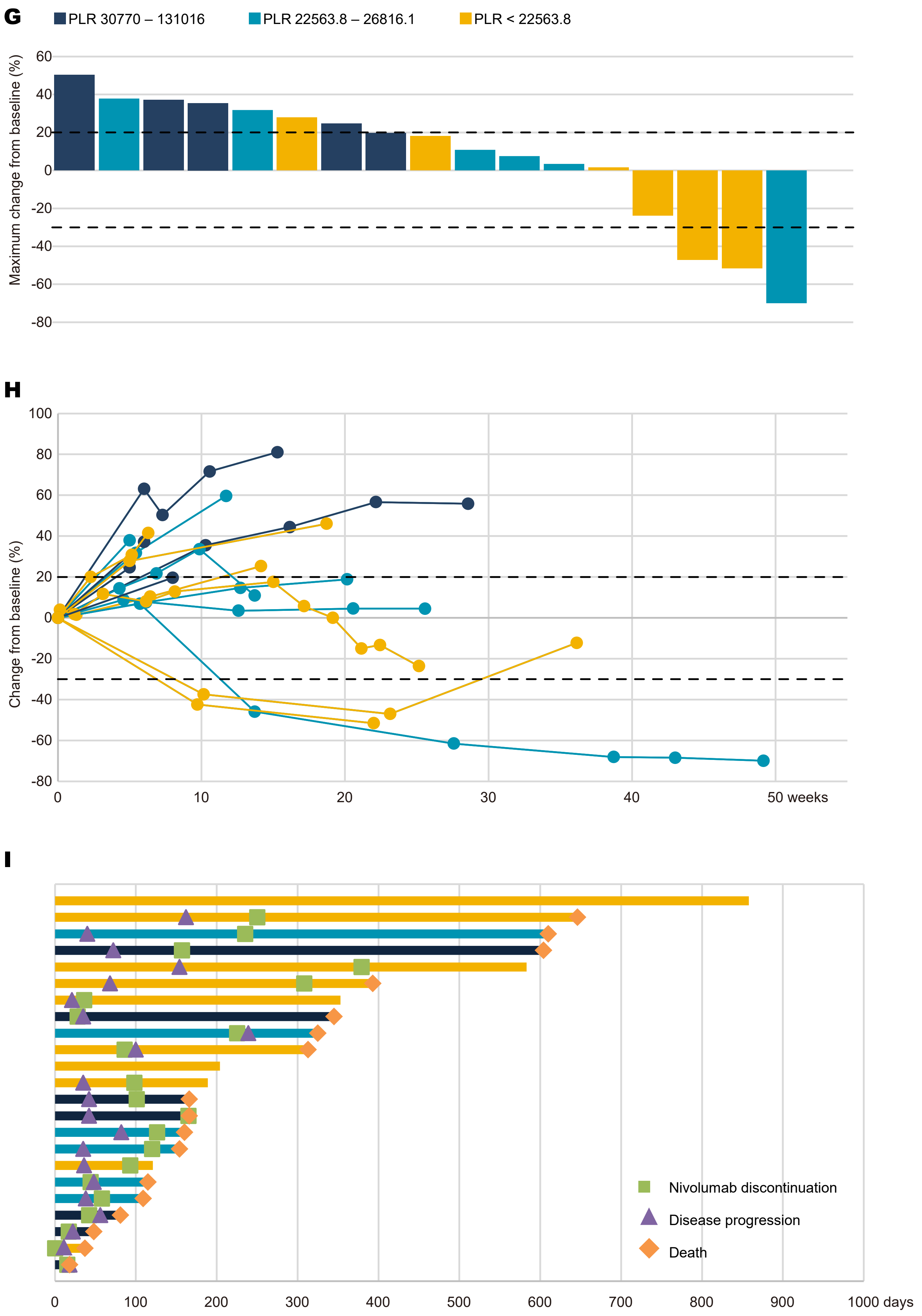
G
PLR 30770 – 131016
PLR 22563.8 – 26816.1
PLR < 22563.8
Maximum change from baseline (%)
60
40
20
0
-20
-40
-60
-80
H
Change from baseline (%)
100
80
60
40
20
0
-20
-40
-60
-80
0
10
20
30
40
50 weeks
I
Nivolumab discontinuation
Disease progression
Death
0
100
200
300
400
500
600
700
800
900
1000 days

J

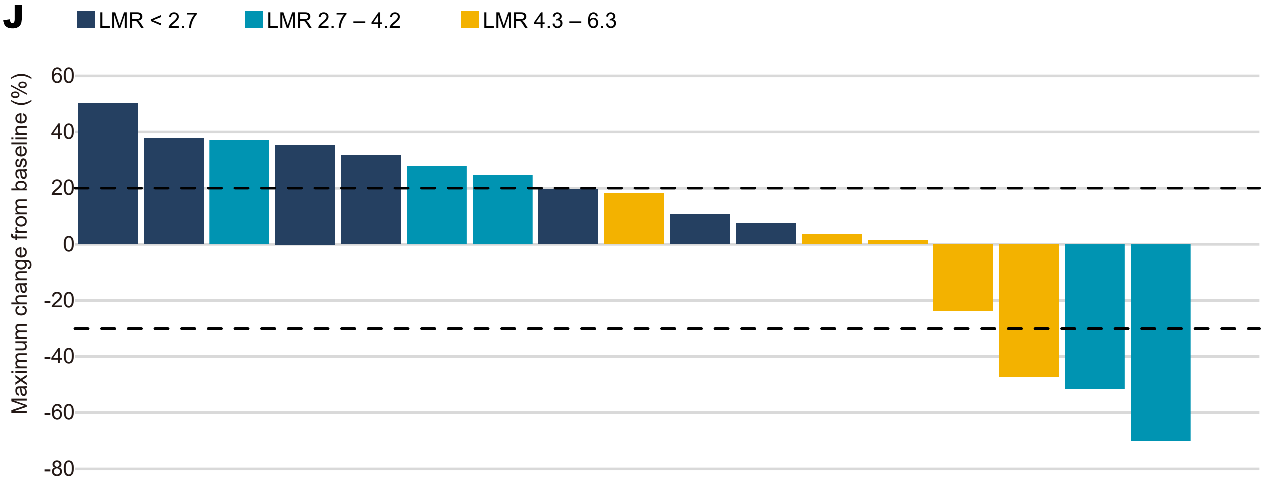
LMR < 2.7
LMR 2.7 – 4.2
LMR 4.3 – 6.3
Maximum change from baseline (%)
60
40
20
0
-20
-40
-60
-80

K

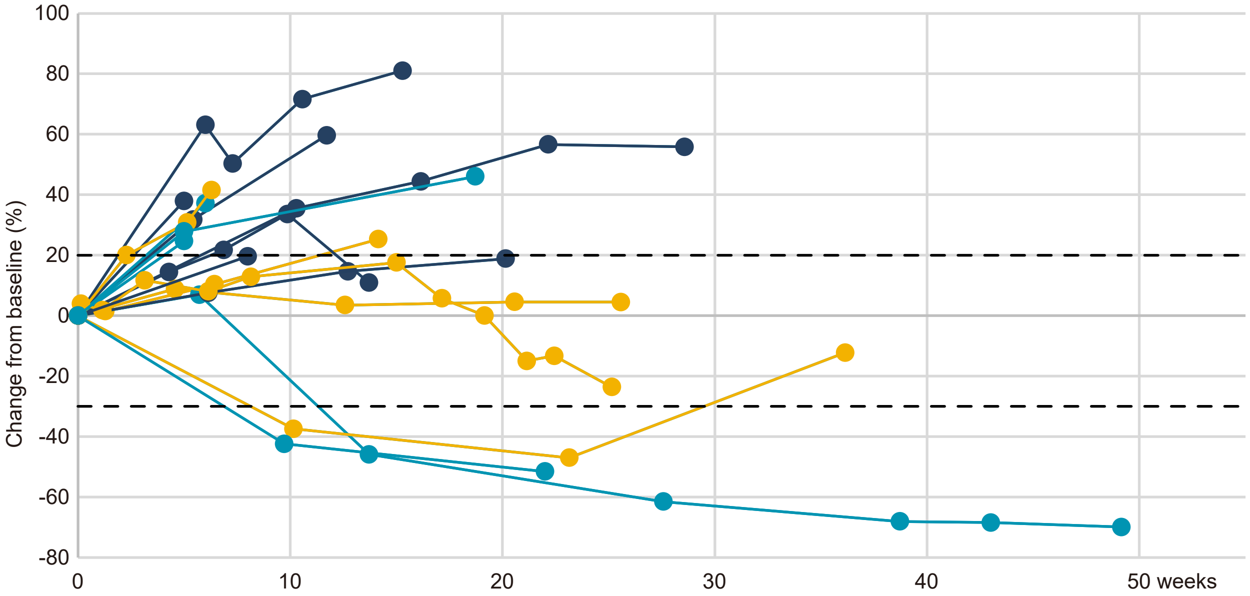
Change from baseline (%)
100
80
60
40
20
0
-20
-40
-60
-80
0
10
20
30
40
50 weeks

L

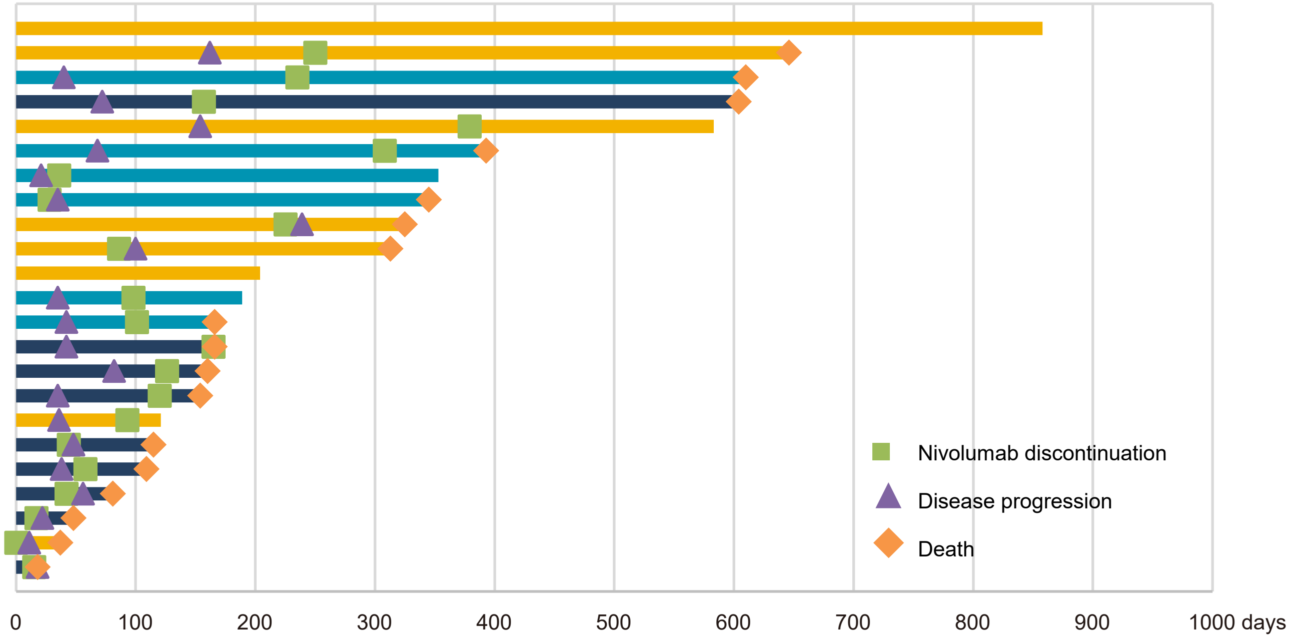
Nivolumab discontinuation
Disease progression
Death
0
100
200
300
400
500
600
700
800
900
1000 days

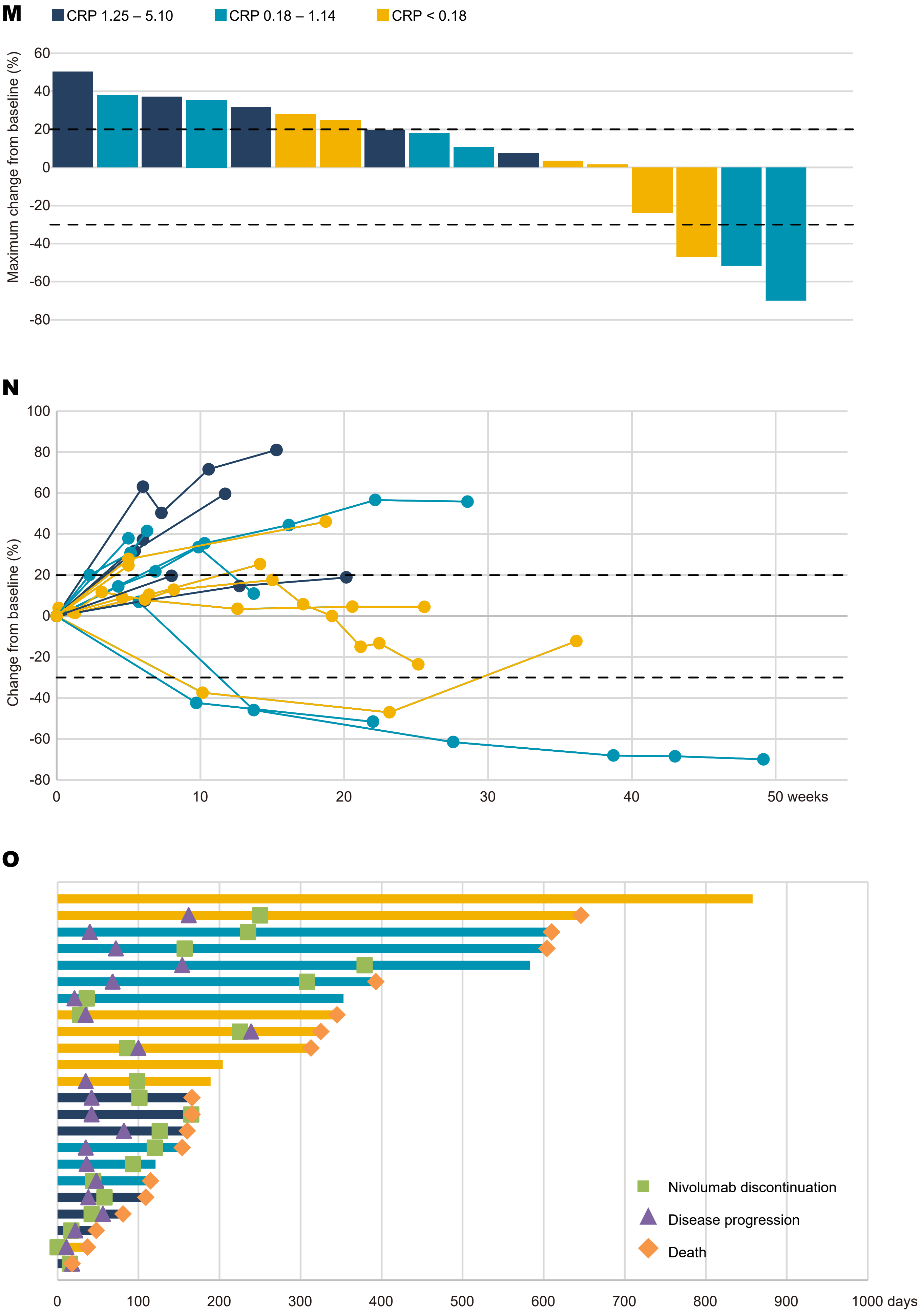
M
CRP 1.25 – 5.10
CRP 0.18 – 1.14
CRP < 0.18
Maximum change from baseline (%)
60
40
20
0
-20
-40
-60
-80
N
Change from baseline (%)
100
80
60
40
20
0
-20
-40
-60
-80
0
10
20
30
40
50 weeks
O
Nivolumab discontinuation
Disease progression
Death
0
100
200
300
400
500
600
700
800
900
1000 days

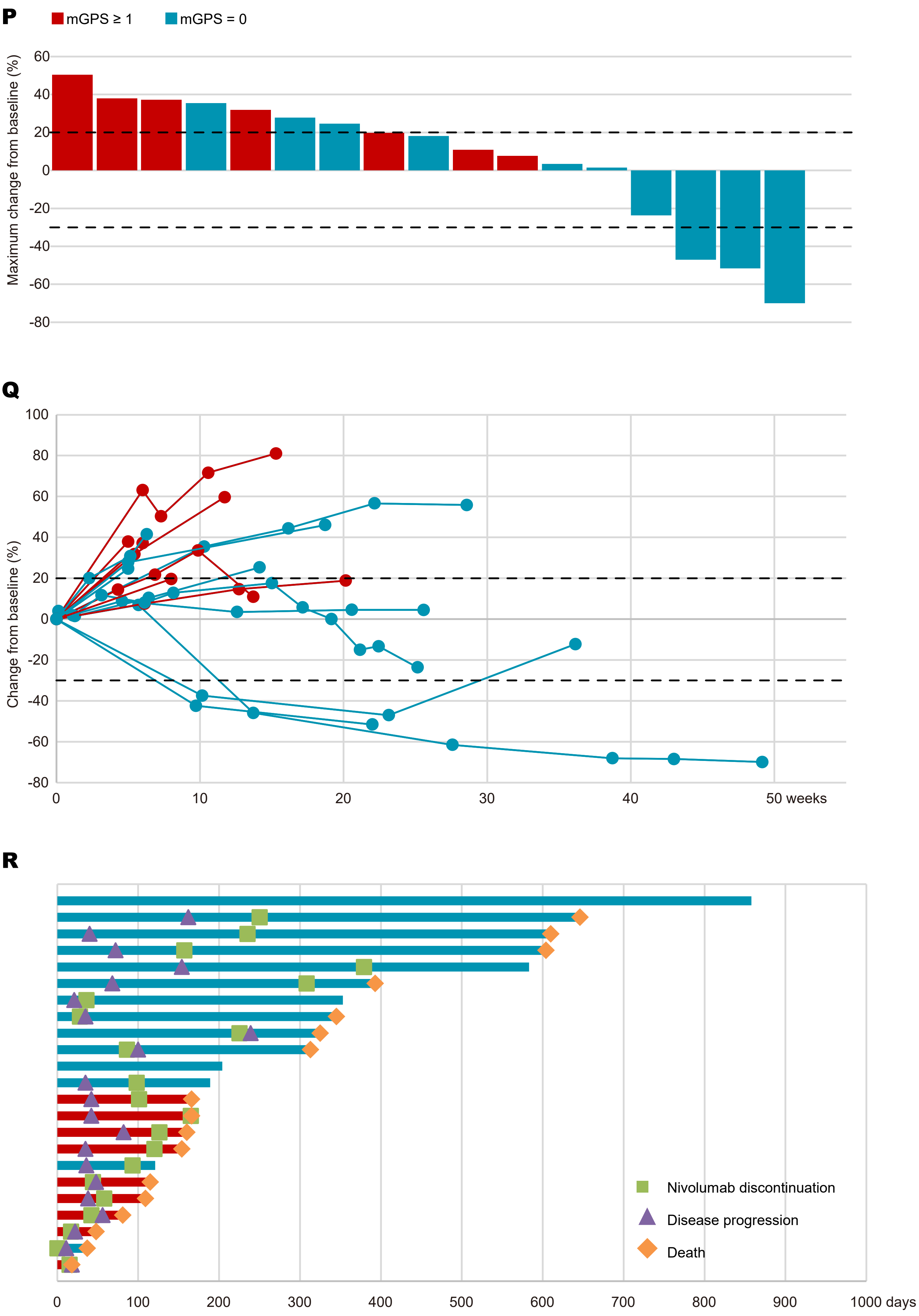
P
mGPS ≥ 1
mGPS = 0
Maximum change from baseline (%)
60
40
20
0
-20
-40
-60
-80
Q
Change from baseline (%)
100
80
60
40
20
0
-20
-40
-60
-80
0
10
20
30
40
50 weeks
R
Nivolumab discontinuation
Disease progression
Death
0
100
200
300
400
500
600
700
800
900
1000 days

S

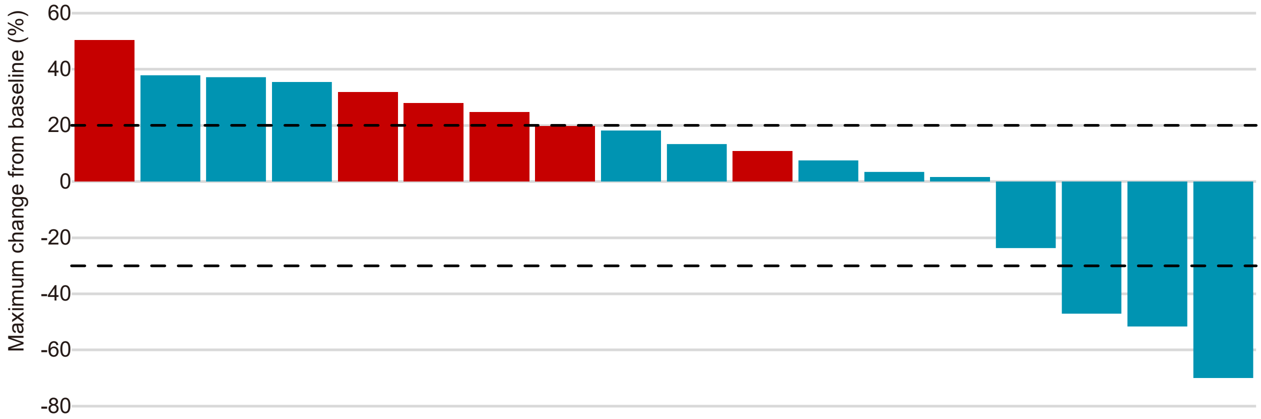
LDH 236 – 586
LDH 118 – 211
Maximum change from baseline (%)
60
40
20
0
-20
-40
-60
-80

T

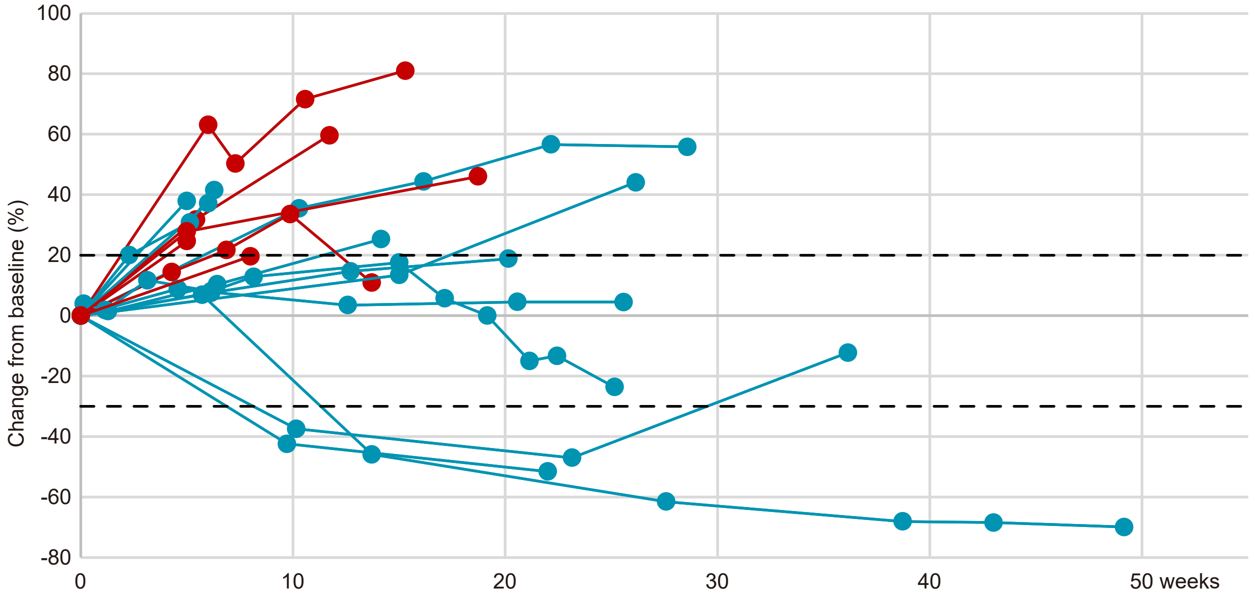
Change from baseline (%)
100
80
60
40
20
0
-20
-40
-60
-80
0
10
20
30
40
50 weeks

U

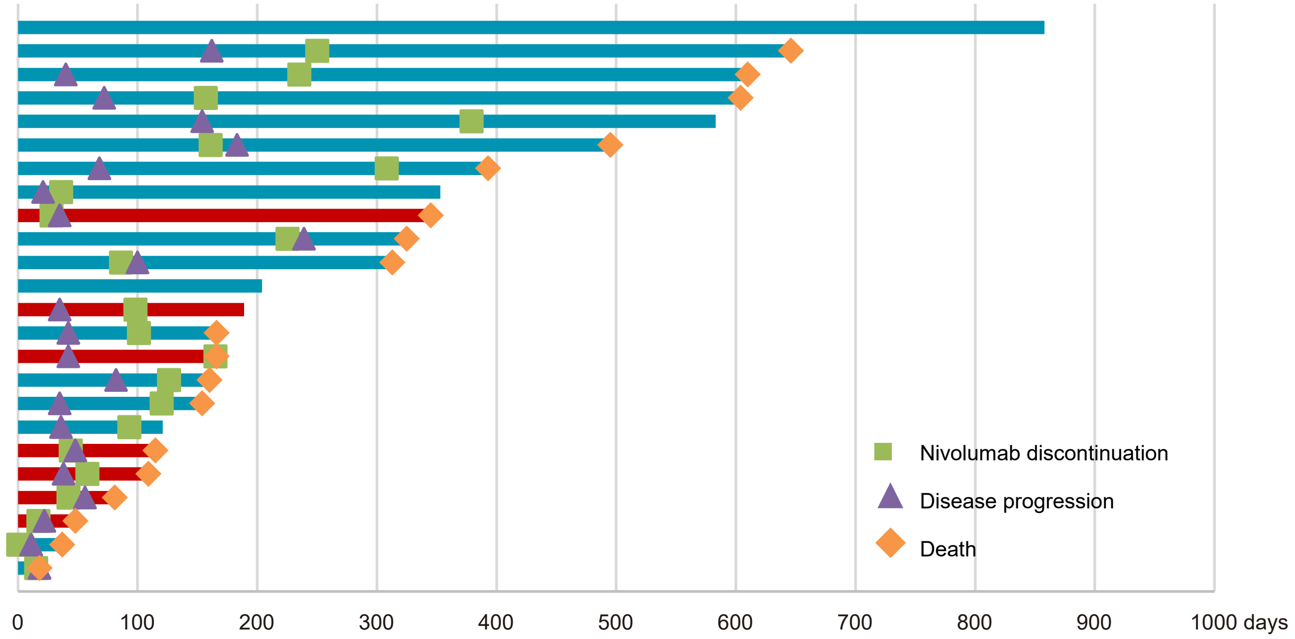
Nivolumab discontinuation
Disease progression
Death
0
100
200
300
400
500
600
700
800
900
1000 days
